# Site-specific PEGylation of micro-plasmin for improved thrombolytic therapy through engineering enhanced resistance against serpin mediated inhibition

**DOI:** 10.1371/journal.pone.0217234

**Published:** 2019-05-29

**Authors:** Navneet Kaur, Prakash Kumar Sinha, Girish Sahni

**Affiliations:** 1 CSIR-Institute of Microbial Technology, Chandigarh, India; 2 Panjab University, Chandigarh, India; Tecnologico de Monterrey, MEXICO

## Abstract

The relatively rapid inhibition of microplasmin by α_2_-AP leads to short functional half-life of the molecule *in vivo*, causing inefficient clot dissolution, even after site-specific, local catheter-based delivery. Here, we describe a PEGylation approach for improving the therapeutic potential via improving the survival of microplasmin in presence of its cognate inhibitor, α_2_-AP, wherein a series of strategically designed cysteine analogs of micro-plasminogen were prepared and expressed in *E*. *coli*, and further modified by covalent grafting *in vitro* with PEG groups of different molecular sizes so as to select single or double PEG chains that increase the molecular weight and hydrodynamic radii of the conjugates, but with a minimal discernible effect on intrinsic plasmin activity and structural framework, as explored by amidolytic activity and CD-spectroscopy, respectively. Interestingly, some of the purified PEG-coupled proteins after conversion to their corresponding proteolytically active forms were found to exhibit significantly reduced inhibition rates (up to 2-fold) by α_2_-AP relative to that observed with wild-type microplasmin. These results indicate an interesting, and not often observed, effect of PEG groups through reduced/altered dynamics between protease and inhibitor, likely through a steric hindrance mechanism. Thus, the present study successfully identifies single- and double-site PEGylated muteins of microplasmin with significantly enhanced functional half-life through enhanced resistance to inactivation by its *in vivo* plasma inhibitor. Such an increased survival of bioactivity *in situ*, holds unmistakable potential for therapeutic exploitation, especially in ischemic strokes where a direct, catheter-based deposition within the cranium has been shown to be promising, but is currently limited by the very short *in vivo* bioactive half-life of the fibrin dissolving agent/s.

## Introduction

The formation of pathological thrombi in the circulatory system can produce significant unwanted consequences like embolism, ischemia, heart attack, stroke, etc. Currently available thrombolytic treatments using plasminogen activators are associated with high cerebral bleeding risks and a 2–3 h, narrow therapeutic time-window especially in case of ischemic stroke [1–4].

‘Direct-acting’ thrombolytic agents such as plasmin and its derivatives (e.g., microplasmin, miniplasmin, delta-plasmin) possess potential for ameliorated thrombolytic therapy with enhanced hemostatic safety [5]. These are potent serine proteases involved in clot dissolution and are intrinsic in origin. Micro-plasmin(ogen) (~29 kDa) is a recombinant truncated form of plasmin(ogen), and consists of only the functionally active catalytic domain. The cleavage of scissile peptide bond, Arg561-Val562 of human plasminogen/micro-plasmin(ogen) by streptokinase or other activators leads to the proteolytic activation of the substrate proteins [6]. α_2_-AP (plasma concentration of ~1μM) and α_2_-macroglobulin (plasma concentration of ~3.5μM) are the main physiological inhibitors of microplasmin [7]. However, the ability of α_2_-macroglobulin to inhibit plasmin is much lower than that of α_2_-AP [8] and it is α_2_-antiplasmin that is believed to be the key player in the fibrinolytic system. It is the fast-acting serpin inhibitor of plasmin which forms a 1:1 stable complex with plasmin, either in the circulation or on the fibrin surface [9]. Lysine-binding sites on plasmin kringles are responsible for its binding to fibrin and its physiologic inhibitor, α_2_-antiplasmin [10–12]. The latter inactivation is among the fastest protein–protein reactions [13].

The effects and safety profile of plasmin has been evaluated in several studies supported by *in vivo* evidences [14–18]. When administered systemically, plasmin is rapidly neutralized within seconds by circulating α_2_-antiplasmin (α_2_-AP) and does not effectively dissolve the thrombus while also certainly not inducing hemorrhagic complications. It was concluded therefore that intravenous plasmin for thrombolytic therapy was safe but was not fully effective as yet for clot dissolution [10, 14, 18–22].

Like plasmin, its truncated version microplasmin also shows remarkable hemostatic safety in various pre-clinical acute stroke models suggesting that, like native plasmin, microplasmin poses significantly less systemic hemorrhagic risk than *rt*-PA [2, 23, 24]. Local delivery of microplasmin induces approximately 50% clot lysis in extracorporeal loop thrombosis model in rabbits and reduces focal cerebral infarction in mice ischemic stroke model [25]. But a very short half-life of microplasmin due to rapid α_2_-AP inactivation fails to effectively dissolve the thrombus even though the risk of inducing hemorrhagic complications remains low.

Interestingly, microplasmin is inactivated at a lower rate than intact plasmin, besides having comparable potency with plasmin, in terms of clot dissolution *per se* [25], likely due to the absence of the lysine binding sites present in the full length protein [25]. The second-order rate constant of microplasmin inhibition by α_2_-antiplasmin is 2×10^5^mmol L^-1^ s^-1^, which is approximately 100 times slower than the inhibition rate of intact plasmin by α_2_-antiplasmin. The lower second order rate constant, corresponds to a half-life of microplasmin in circulating blood of approximately 4 s, as compared to a half-life of 0.02 s for plasmin [25] but is still short of the requirements for successful therapy. However, owing to its primary attributes such as intrinsic origin, self-sustaining mechanism (plasminogen-independent action) and neuro-protective nature combined with the advantages of easy production and slow rate of inhibition over intact plasmin, microplasmin has been proposed as a better futuristic agent to treat thrombotic disorders [4]. Nevertheless, possessing a higher efficacy and positive α_2_-AP safety profile, if the relatively rapid inhibition of microplasmin by α_2_-AP is thwarted, will help in its successful development as an effective thrombolytics. In other words, there exists an urgent need for designing more efficacious mutants of microplasmin with improved survival against inhibition by the cognate blood serpin, α_2_-antiplasmin.

This report attempts to address these issues by employing a site specific PEGylation approach. Protein-PEGylation is an established method to modulate the molecular interactions and enhance circulation half-life of protein-based therapeutics [26–31]. PEG (Polyethylene glycol) has been approved by the Food and Drug Administration (FDA) [32]. The covalent coupling of polyethylene glycol (PEG) to proteins enhances their hydrodynamic size and shields protein sites from recognition by the immune system, cellular receptors, or proteases [33–38]. These properties lead to decreased renal, enzymatic, and cellular clearance, resulting in prolonged circulation half-lives in the bloodstream [39, 40]. Thus, PEGylated proteins and peptides have been very useful as improved therapeutic agents in recent years [41]. It has been shown that coupling of PEG polymer is able to reduce protein–protein interactions between therapeutics, proteins, and cells *in vivo* [42]. PEGylation of cofactor altered the interactions between the enzyme and modified cofactors by affecting the rate of formation of enzyme/cofactor complexes and/or the formation of enzyme/cofactor/substrate complexes [43]. Diverse studies agree that PEG has substantial effect on structural dynamics and stability of proteins such as α-chymotrypsin [44], insulin [45], lysozyme [46, 47] etc. These effects are typically attributed to a protective shielding of PEG wrapped around the protein. Numerous strategies are available for coupling of PEG moieties to one or more residues on the protein or peptide [48]. Although the technique of PEG-coupling is generic, but discreet positioning of PEG moiety in a therapeutic protein is eminently important. Generally, it is known that properties such as biological activity and half-life of conjugated protein/s depend on the site of modification and the size of PEG-groups involved [48, 49]. The development of selective site modification reactions through a thiol-based chemistry has enabled researchers to PEGylate proteins with high selectivity at defined interfaces [50]. PEGylation of cysteine mutants of staphylokinase, GCSF, streptokinase and several other therapeutic proteins has resulted in enhanced circulatory half-life while maintaining their primary potency [51–53].

In the present study, we describe PEG-decorated microplasmins with a significant protection from the rapid inactivation observed with unmodified microplasmin by its plasma inhibitor, which are attractive as leads for further therapeutic testing.

## Materials and methods

### Materials

The micro-plasminogen (truncated plasminogen derivative) previously cloned in T7 RNA polymerase inducible promoter based expression vector pET11a was obtained from lab [54]. Expression host *E*. *coli* strain BL21(DE3) was procured from Novagen Inc. (Madison, Wisconsin, USA). All the oligonucleotide primers used in the study for mutagenesis were custom synthesized from the Integrated DNA Technologies (IDT), USA. Methoxy-PEG maleimide reagent was purchased from JenKem Technology, USA. All the materials required for the SDS-PAGE were purchased from *Bio-RAD*, USA. Commercially available α₂-Antiplasmin from Calbiochem was used for plasmin inhibition kinetics. Chromozym PL was purchased from Roche Diagnostics, USA. SP-Sepharose and Superdex-75 pg matrix used for different chromatographic processes were procured from Pharmacia Amersham-GE, Uppsala, Sweden. Zeba Spin Desalting Columns were purchased from Thermo Fisher Scientific, USA. Spectrophotometric analysis was done using Perkin Elmer LAMBDA 35 UV/Vis spectrophotometer. All the reagents used were of the highest analytical grade available.

### Methods

#### Expression and purification of recombinant micro-plasminogen

Micro-plasminogen previously cloned in *E*.*coli* [54] was obtained after IPTG (isopropyl-thiogalactopyranoside) induction in the form of inclusion bodies, which were then solubilised in 8M urea and 10mM DTT. The denatured and reduced protein was further subjected to *in vitro* refolding using refolding buffer (50mMTris-Cl pH 8.0, 1mM EDTA, 1.6M urea, 20% glycerol, 1.25mM GSH and 0.5mM GSSG) for 48 h at 4°C. Refolded micro-plasminogen was purified by cation-exchange chromatography on SP-Sepharose (GE-Amersham Biosciences). The protein eluted with 1M NaCl in 20mM Sodium acetate buffer (pH 5.5) was further desalted in 50mM PB (pH7.4) using Zeba desalting columns.

#### Design, construction and purification of microplasmin mutants

The residues for PEGylation were selected on the basis of surface accessibility and likelihood of association with α_2_-AP using available structural information. The available three dimensional structural information of murine antiplasmin as well as human plasminogen catalytic domain [55] and their docking models obtained from GRAMM-X Protein-Protein Docking Web Server v.1.2.0 [56] were used to interpret interacting loci between these two proteins. PDBePISA software was used to analyze the solvent accessibility of the selected residues of micro-plasminogen (PDB 1ddj).

Single-site as well as double-site cysteine mutants of micro-plasminogen were constructed using site-directed mutagenesis (QuickChange mutagenesis kit obtained from Stratagene Inc., WI, USA). By the use of *pfu* turbo enzyme, both plasmid strands were replicated with high fidelity using two complementary primers having the desired mutation. The parental plasmid was digested with DpnI (Thermo Fisher Scientific, USA) restriction enzyme that cleaves specifically methylated DNA [57]. The plasmid was then transformed into *E*.*coli* XL1-Blue competent cells to obtain transformants which were further validated by DNA sequencing. All the cysteine variants were expressed as inclusion bodies, refolded and purified by cation-exchange chromatography by following the same methodology as used for wild-type micro-plasminogen. Protein concentrations were determined by Bradford reagent and further confirmed by measuring the UV absorbance at 280nm.

#### Thiol estimation and PEGylation of micro-plasminogen mutants

The number of free thiols in cysteine mutant proteins was estimated by a classical colorimetric method using Ellman’s reagent *viz*.5,5′ -dithiobis (2-nitrobenzoic acid). Beta-mercaptoethanol having a single free functional thiol, was used as standard [58]. Following the validation of the present free thiol/s in each mutant, the proteins were incubated with 15–20 fold molar excess of malemide-activated linear methoxy PEG (JenKem Technology, USA) of different molecular weights (e.g. 20kDa and 40kDa) in the presence of 100mM Tris-Cl (pH 8) and 2mM EDTA. The reaction mixtures were allowed to gently stir for 3 h at room temperature.

#### Purification and analysis of modified micro-plasminogen analogs

The PEGylation reaction mixture was further desalted with 20mM sodium acetate (pH 5.5) using Zeba Spin Desalting Columns (Thermo Fisher Scientific, USA). The mixture was then loaded onto a SP-Sepharose column pre-equilibrated in 20mM sodium acetate, pH 5.5 with a flow rate of 2ml/min. The bound protein was eluted using linear gradient of 1M NaCl. The eluted protein fraction was further purified to obtain more uniform PEGylated product using a Superdex-75 pg (16 × 600 mm) size exclusion chromatography to separate un-reacted protein fraction from the PEGylated protein. SP-Sepharose purified protein was concentrated and then injected into a Superdex-75 pg (16 × 600 mm) column using a 1ml sample loop. The column was run at a flow rate of 0.5ml/min. All the purifications were performed at 4°C using an ÄKTA Purifier system (GE Healthcare Life Sciences, USA).

#### Analytical characterization of PEGylated micro-plasminogen analogs

All PEGylated as well as un-PEGylated derivatives were checked for their purity on SDS-PAGE. Quantitative amino acid composition analysis of PEGylated mutants was performed using a Waters Pico-Tag HPLC Amino Acid Analysis System. Further structural and functional studies were performed with ~99% purified mono-PEGylated/ di-PEGylated proteins.

#### Mass spectrometry analyses

The mass values of all the analogs were determined by matrix-assisted laser desorption ionization time of flight mass spectrometry (MALDI-TOF) on an ABISCIEX machine, TripleTOF 5600/5600.

#### Circular dichroism spectroscopy

CD analysis was performed to investigate the secondary structure of micro-plasminogen derivatives upon PEGylation. Far-UV CD spectra of wild-type as well their modified analogs were recorded from 195-250nm on Jasco J-815 spectropolarimeter at 25°C. Measurements of all the samples at concentration 0.2mg/ml, were carried out using cuvette of 0.1 cm path length.

#### Hydrodynamic size measurements

To determine the hydrodynamic sizes of the PEGylated and un-PEGylated micro-plasminogen analogs, dynamic light scattering (DLS) analyses were performed on Nano Z (Malvern Panalytical, UK) instrument. The measurements were carried at 25°C with sample concentration of 0.5-1mg/ml in phosphate buffer. Data acquisition and cumulant analysis of runs in triplicate was done by using Zetasizer software.

#### Activation and amidolytic activity determination

The purified mono-PEGylated as well as di-PEGylated thiol derivatives of micro-plasminogen were converted to their active forms using urokinase-coupled Sepharose beads in presence of 50mM Tris-Cl (pH 8), 25mM lysine and 25% (v/v) glycerol. The reaction was set up at 22°C with slow stirring for up to 8–10 h and monitored at regular intervals using Chromozym PL [59, 60].

The kinetic parameters of microplasmin analogs for amidolytic activity were determined by measuring the cleavage of the para-nitroanilide peptide substrate at 405nm using [59]. Varying concentrations of the chromogenic substrate, namely Chromozym-PL (tosyl-Gly-L-ProL-Lys-pNA) (0.1 to 4mM) were added to each microplasmin variant (final conc. 20nM) in presence of assay buffer (50mM Tris-Cl, pH 7.4,100 mM NaCl). The reaction was continuously monitored spectrophotometrically at 37°C for 10 min. The data were plotted as V/S and analyzed by hyperbolic curve fitting using a Sigma Plot program [61, 62].

#### Fibrin plate assays

*In vitro* biological activity of activated PEGylated micro-plasminogen analogs was performed by fibrin plate method [63]. Briefly, fibrin plates were prepared by adding fibrinogen (1.2mg/ml) and thrombin (0.3 NIH units/ml) in a 2% agarose solution. The solution in the petri dish was left for 30 min at room temperature to form a fibrin clot layer. 20μl of activated samples (0.5mg/ml) were added to the wells (3mm diameter) and incubated at 37°C for 10 to 20 h. The fibrinolytic activity was qualitatively estimated by the zone of hydrolysis.

#### Evaluation of α_2_-AP inhibition of PEGylated microplasmin analogs

α_2_-AP inhibition kinetics was performed by separately adding microplasmin analogs (20nM) and antiplasmin (60nM, Calbiochem) to cuvette containing 100mM sodium phosphate, pH 7.2 and incubating at 25°C for the time interval ranging from 15sec-30 min. Change in absorbance at 405nm was recorded at 60 s intervals after the addition of 0.5mM Chromozym PL. The residual enzyme activity was measured at different intervals from the slope of the curve and plotted as log % residual activity *versus* time [13, 62].

## Results

### Design, construction and purification of micro-plasminogen mutants

**Fig 1:**
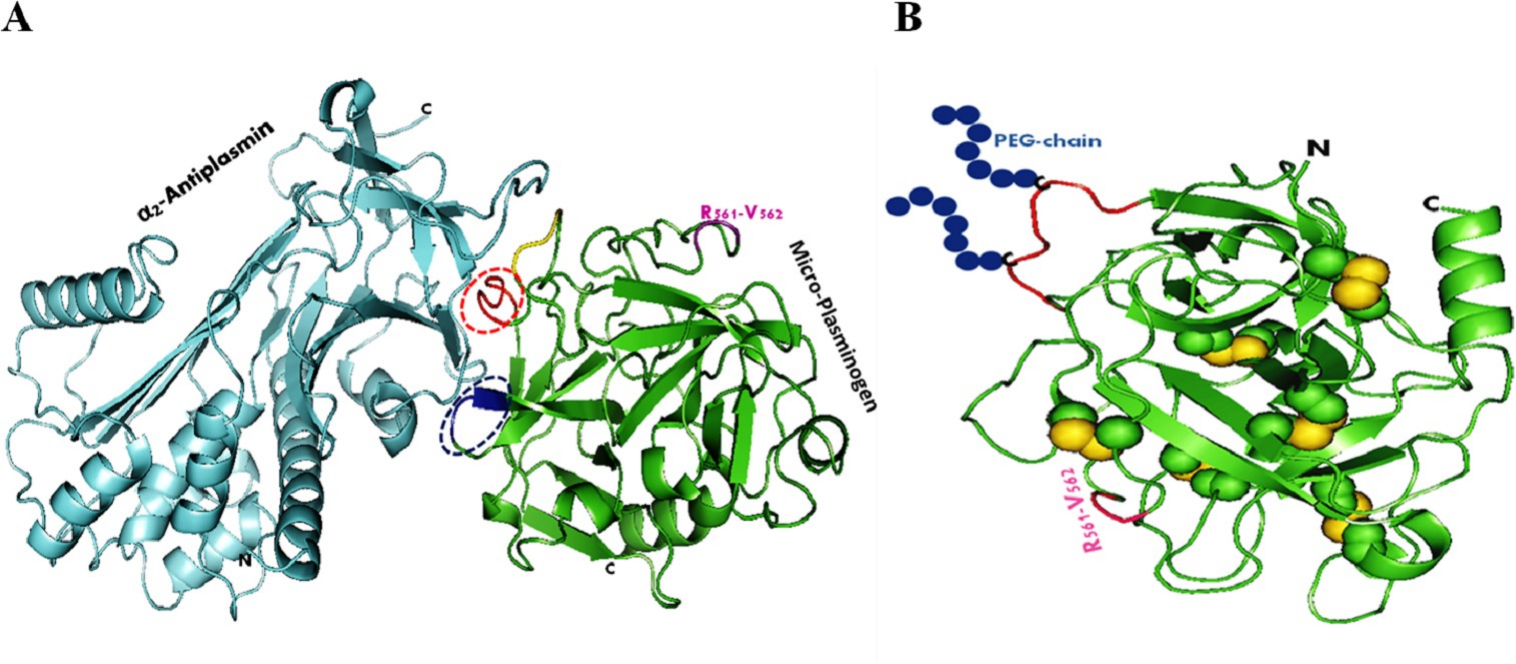
Rationale and scheme of site-specific modification of micro-plasminogen. Ribbon diagrams of the functional domain of human plasminogen are shown in complex form with α_2_-antiplasmin, predicted using GRAMM-X Protein-Protein Docking Web Server v.1.2.0. (A) Fragments (red and blue) in micro-plasminogen structure (green) represent the selected residues for site-specific covalent modification. (B) Blue beads are schematic presentation of PEG polymers attached at one (or more) selected sites on micro-plasminogen.

The catalytic domain of plasminogen comprises of several distinct surface-exposed loops [55]. The loop regions among different serine proteases are considered to be important for their selective interactions with substrates and inhibitors [55, 64]. The X-ray crystal structure of human antiplasmin (α_2_-AP) has not been solved yet. But the crystal structure of murine anti-plasmin, which shares ~78.5% sequence similarity with human plasminogen is known [65]. The predicted 3D structure of micro-plasminogen as complex with murine α_2_-antiplasmin generated by GRAMM-X Protein-Protein Docking Web Server v.1.2.0 (shown in Fig 1) was used to interpret the potential loci of interaction/s between the two proteins. This included a stretch of eight consecutive amino acids, EVNLEPHV (denoted in red in Fig 1A), and three consecutive amino acids, namely FGM (denoted in blue in Fig 1A). Solvent accessibility of these residues was carefully [Table 1] examined. Further, the selected residues were mutated to cysteines, and PEGylated through thiol mediated chemistry after expression of the corresponding genes in *E*. *coli* as described above under Materials and Methods. In Fig 1B, blue beads are schematic presentations of PEG polymers attached at the selected locations on micro-plasminogen.

**Table 1:** Solvent accessible surface area of selected residues (PDBePISA).

Residue	Solvent-Accessible Surface Area, Å^2^
PHE 583	164.96
MET 585	120.62
GLY 584	28.47
GLU 623	19.17
VAL 624	105.58
ASN 625	116.51
LEU 626	54.13
GLU 627	91.35
PRO 628	127.51
HIS 629	64.41
VAL 630	24.61

**Fig 2:**
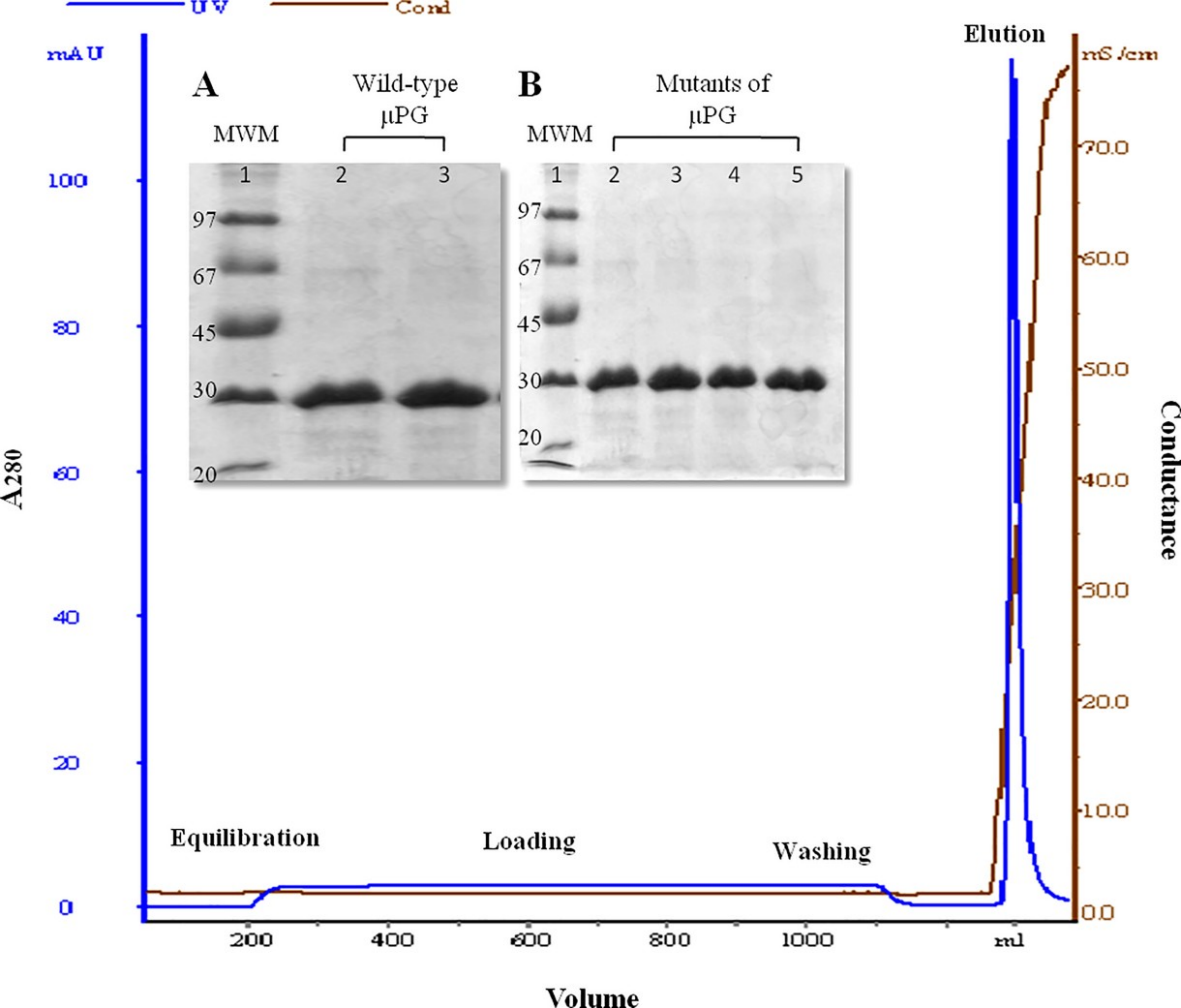
Purification of micro-plasminogen and its thiolated mutants. Cation-exchange chromatography (SP-Sepharose Fast-flow) profile of the wild-type micro-plasminogen is shown here. Protein was purified by gradient elution with 1M NaCl. Parameters such as absorbance at 280nm and conductance have been represented with blue and red line, respectively. Similar chromatograms were obtained for the single- and double-cysteine mutants. The SDS-PAGE pattern shows the general purity of the eluted fractions of wild-type micro-plasminogen (Panel A: Lane 2 and 3) and its mutants (Panel B: Lane 2–5).

Based on predicted sites, eight cysteine analogs including single site as well as double site mutations were constructed (see Material and Methods section). The thiolated micro-plasminogen analogs were further expressed in *E*. *coli* intracellularly, refolded oxidatively from inclusion bodies and purified through a two-step process with an average yield of 8-10mg/L. The SDS-PAGE analysis of purified wild-type micro-plasminogen as well as its cysteine analogs is shown in Fig 2. DTNB assays confirmed the presence of free thiols as per the introduced cysteine/s in the respective micro-plasminogen mutants.

### PEGylation of micro-plasminogen mutants and their purification

**Fig 3:**
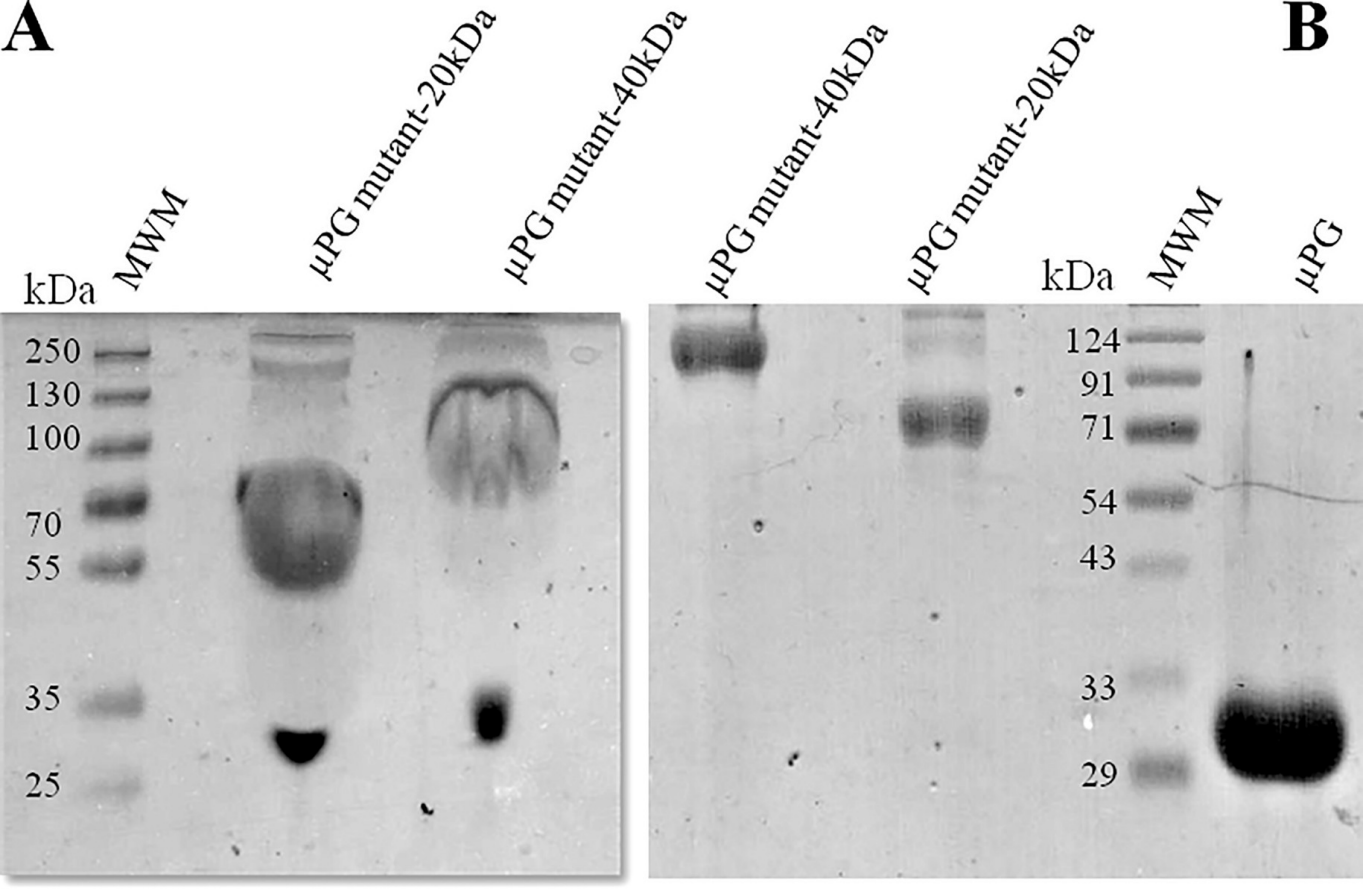
PEGylation of mutant proteins. In panel (A)12%SDS-PAGE profile shows the un-purified reaction products of coupling of PEG groups (20kDa and 40kDa) to cysteine mutants of micro-plasminogen. The corresponding fractions of purified reaction are shown in panel (B) along with the un-PEGylated micro-plasminogen, and standard molecular weight markers.

**Fig 4:**
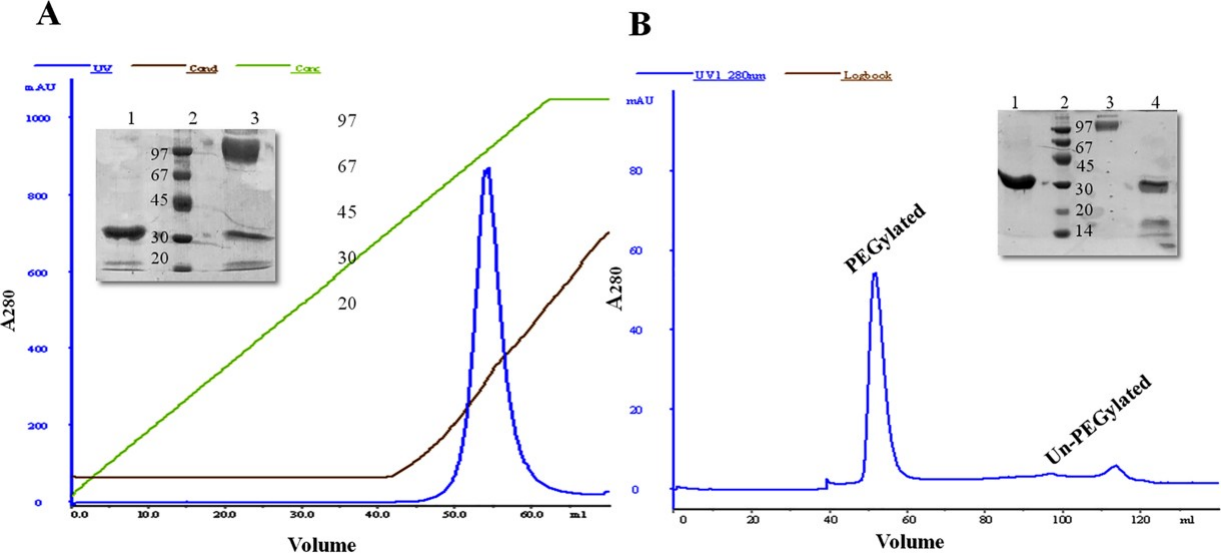
Purification of PEGylated micro-plasminogen derivatives. Panel (A) represents the first-step purification (Cation-exchange chromatography) profile of PEGylated derivatives of micro-plasminogen. SDS-PAGE picture showing, Lane 1. Un-PEGylated micro-plasminogen mutant, 2. Standard marker Protein Ladder, 3. Eluted peak fraction corresponding to PEG-conjugated protein, and the un-reacted part. Panel (B) shows the second-step purification (Size-exclusion chromatography) profile. Lane 1. Un-PEGylated micro-plasminogen mutant, 2. Protein ladder, 3. Peak 1 fraction corresponding to PEG-conjugated protein, 4. Peak 2 fraction corresponding to un-reacted fraction.

Under the optimized non-reducing conditions, PEGylation of the thiol derivatives of micro-plasminogen, exploiting methoxy-malemide chemistry [48, 66], resulted in approximately 75–80% of PEGylated complexes, while 15–20% remained un-PEGylated as observed on SDS-PAGE (see Fig 3). After the conjugation yield had been optimized, the PEGylated derivatives were enriched by a simple two-step purification process, namely cation-exchange chromatography, followed by gel-filtration chromatography. Free (un-reacted) PEG was removed by cation-exchange chromatography. Gel-filtration chromatography yielded two major peaks. The first peak corresponded to mono-PEGylated micro-plasminogen and the later-eluting peak corresponded to unmodified protein as indicated by SDS-PAGE analysis (Fig 4).

### Characterization of PEGylated and un-PEGylated micro-plasminogen

**Fig 5:**
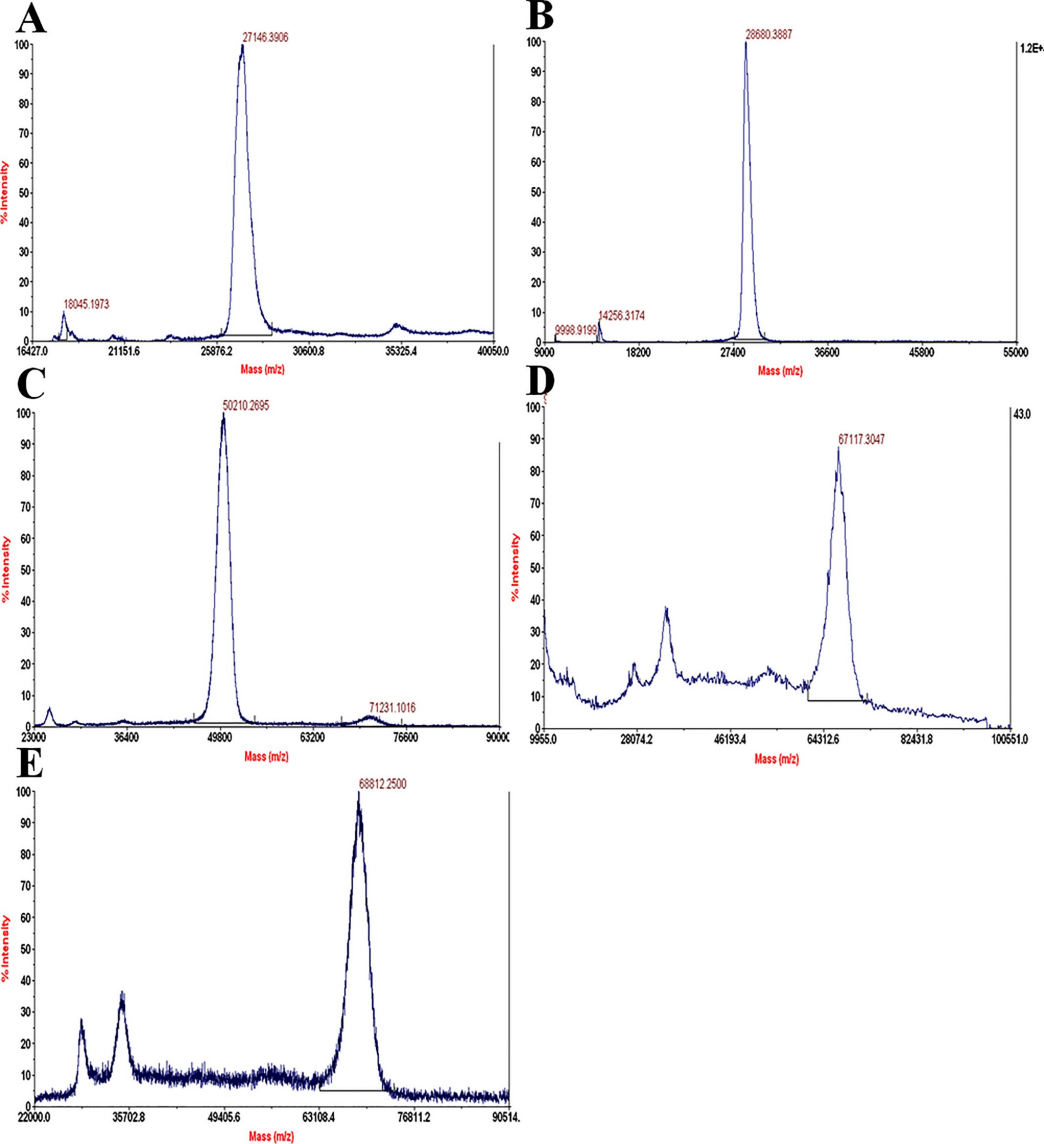
Mass spectral analyses of the micro-plasminogen and its PEGylated mutants. Panel (A) shows the MALDI-TOF peak of wild-type micro-plasminogen (~28kDa). Panel (B) shows the MALDI-TOF peak of one of the cysteine mutant of micro-plasminogen. Similar profiles were obtained in case of other cysteine mutants. Panel (C) represents the MALDI-TOF peak of the purified PEGylated (20kDa) micro-plasminogen mutants. Panel (D) represents MALDI-TOF peak corresponding to mono-PEGylated μPG mutant (40kDa).Panel (E) represents the MALDI-TOF profile of the di-PEGylated mutant of micro-plasminogen comprising two PEG chains of 20kDa each. The molecular masses observed by MALDI-TOF were quite close their theoretical ones.

SDS-PAGE profile of PEG coupling reactions (20kDa and 40kDa) to cysteine mutant of micro-plasminogen shows that, PEGylation reaction yielded near-homogeneous covalently modified plasminogen derivatives (Figs 3 and 4). However, mono-PEGylated as well as di-PEGylated variants tend to migrate at a higher apparent molecular weight than the one predicted from the sum of the molecular weights of both protein and PEG group. This anomalous behavior of PEG is by virtue of its large hydrodynamic volume [67], which causes retarded electrophoretic mobility of PEG-conjugated proteins. MALDI-TOF data of the wild-type micro-plasminogen and its PEGylated analogs (shown in Fig 5), confirmed their size, which were close to expected theoretical values [Table 2].

**Table 2:** Molecular mass of micro-plasminogen and its PEGylated mutants.

Micro-plasminogen variant	SDS-PAGE(kDa)	MALDI-TOF (kDa)	Calculated (kDa)
Recombinant wild-type μPG	~29	28.68	27.231
mono-PEGylated μPG mutant (20kDa)	~71	50.21	47.231
mono-PEGylated μPG mutant (40kDa)	~97	67.12	67.231
di-PEGylated μPG mutant (20kDa-20kDa)	~97	68.81	67.231

**Fig 6:**
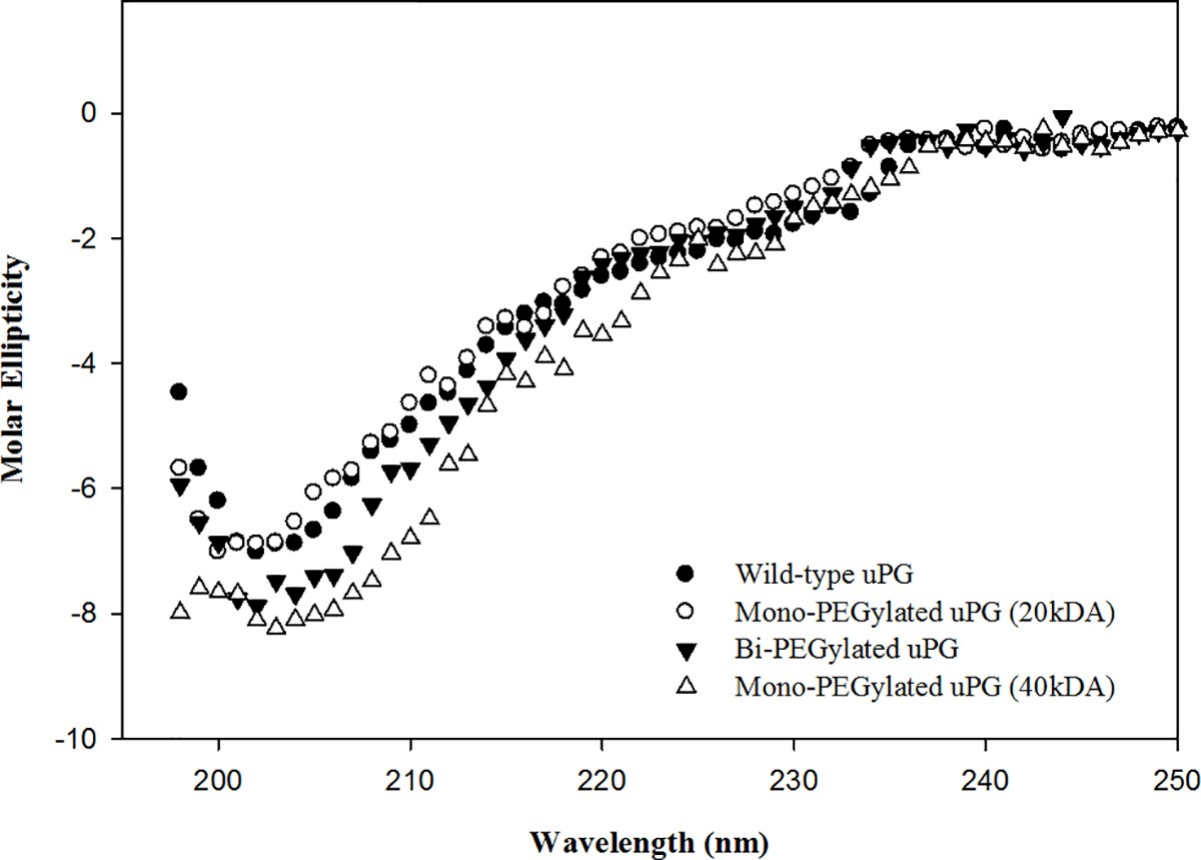
Far UV Circular Dichroic spectra of PEGylated micro-plasminogen mutants. Circular dichroism analysis was carried out for mono-PEGylated and di-PEGylated cysteine analogs as well as un-PEGylated micro-plasminogen. Spectra were recorded at 195-250nm at 25°C with the samples concentration 0.2mg/ml. Apart from very minor changes in the far UV region (around 190-200nm), the CD spectra indicate that the overall native like secondary structures were preserved in the mutants, in keeping with their essentially native like amidolytic activities (see below).

Far-UV CD spectra (Fig 6), obtained for PEGylated micro-plasminogen almost superimposed with that of wild-type micro-plasminogen, demonstrating that the native-like secondary structure of micro-plasminogen-PEG conjugates were maintained through the coupling reactions and subsequent purifications.

Furthermore, the hydrodynamic radii of micro-plasminogen samples were measured by dynamic light scattering (DLS). It was observed that conjugation with PEG groups has significantly expanded the hydrodynamic radii of the protein sample. As shown in Table 3, the molecular radii of PEGylated microplasmin analogs were higher than that of un-PEGylated microplasmin.

**Table 3:** Hydrodynamic size measurements.

Construct	R_h_, Hydrodynamic radius (nm)
Micro-plasminogen	2.6
mono-PEGylated μPG mutant (20kDa)	5.8
mono-PEGylated μPG mutant (40kDa)	6.4
di-PEGylated μPG mutant (20kDa-20kDa)	4.7

### Evaluation of amidolytic parameters

Kinetic studies were performed to determine the active site integrity of PEG conjugated analogs of microplasmin (see Materials and methods for details). Kinetic parameters of PEGylated microplasmin analogs were found to be comparable to the un-PEGylated microplasmin [Table 4]. However, there was slight increase in Km values for amidolytic substrate over that of un-PEGylated microplasmin which can be accounted for slightly reduced accessibility.

**Table 4:** Amidolytic parameters of microplasmin and its PEGylated derivatives.

Construct	Amidolytic Parameters		
	Km μ*M*	*kcat* *s⁻*^*1*^	*kcat*/Km μ*M ⁻*^*1*^ *s⁻*^*1*^
Micro-plasminogen	**2013±201**	**18 ±0.8**	**0.008**
mono-PEGylated μPG mutant (20kDa)	**2290±254**	**23.45 ±2.5**	**0.010**
mono-PEGylated μPG mutant (40kDa)	**2519±430**	**26.05 ±2.5**	**0.010**
di-PEGylated μPG mutant (20kDa-20kDa)	**2310±220**	**21.32 ±4.5**	**0.009**

### Fibrin plate assay

**Fig 7:**
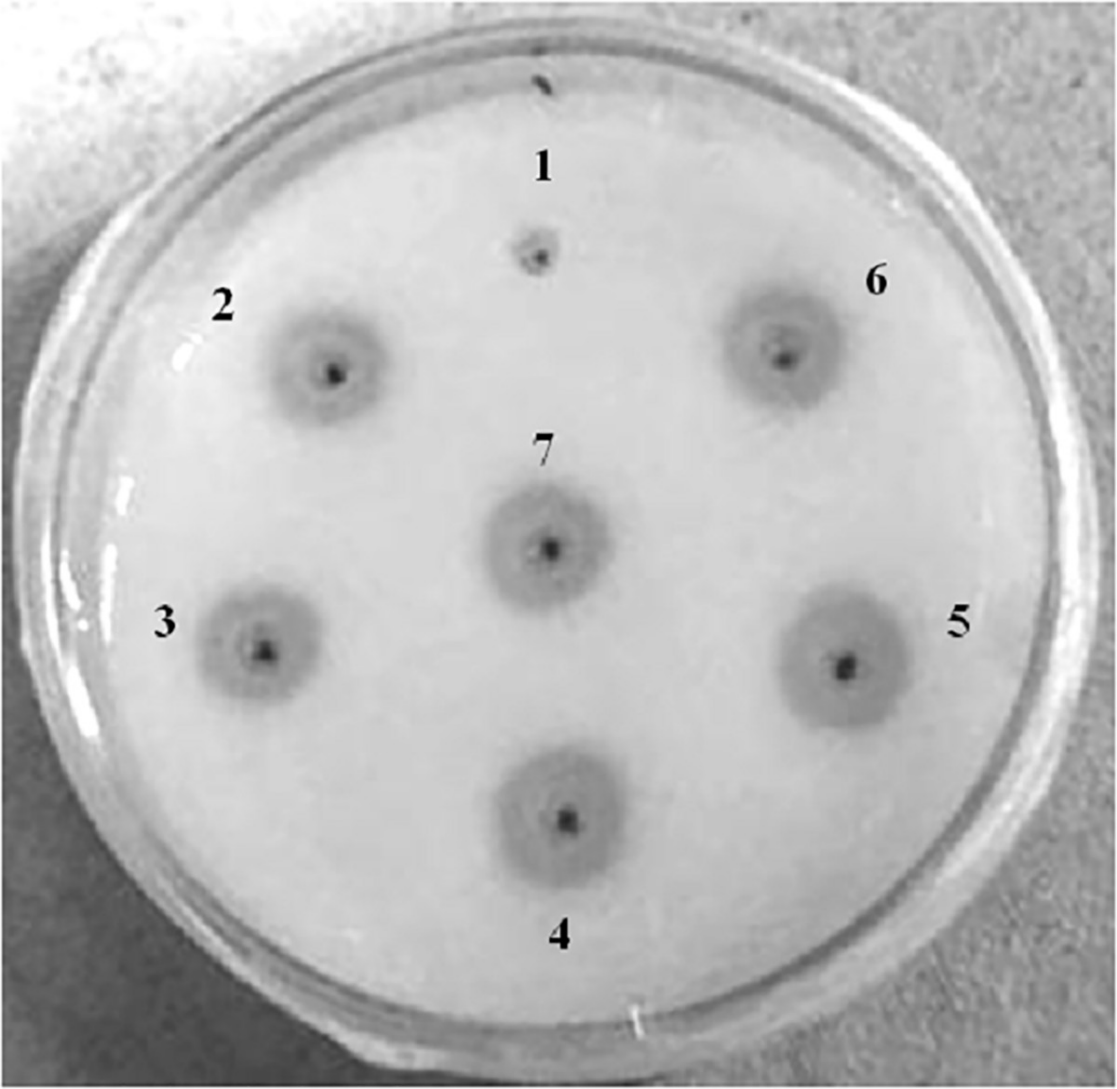
*In vitro* bioactivities of activated PEGylated micro-plasminogen analogs. Fibrin plates were prepared by adding fibrinogen (1.2 mg/ml) and thrombin (0.3 NIH units/ml) in a 2% agarose solution. 20μl of activated samples (0.5mg/ml) were added to the wells (~mm-diameter) and incubated at 37°C for 10 to 20 h as described under “Materials and methods”. In plate, well 1. Buffer (50mM PB pH 7.4), 2. mono-PEGylated μPN-40kDa, 3. mono-PEGylated μPN-20kDa, 4. Wild-type μPN, 5. di-PEGylated μPN-20kDa (each), 6. Un-PEGylated μPN-mutant, 7. Native human Plasmin.

Activated protease forms of PEGylated mutants were tested for their fibrinolytic activity by a classical qualitative approach. mono-PEGylated as well as di-PEGylated mutants of micro-plasminogen showed zone of hydrolysis on fibrin plates (Fig 7). It was observed that fibrinolytic activity was preserved after PEG conjugation.

### *In vitro* inhibition studies of microplasmin mutants by alpha 2-Antiplasmin

**Fig 8:**
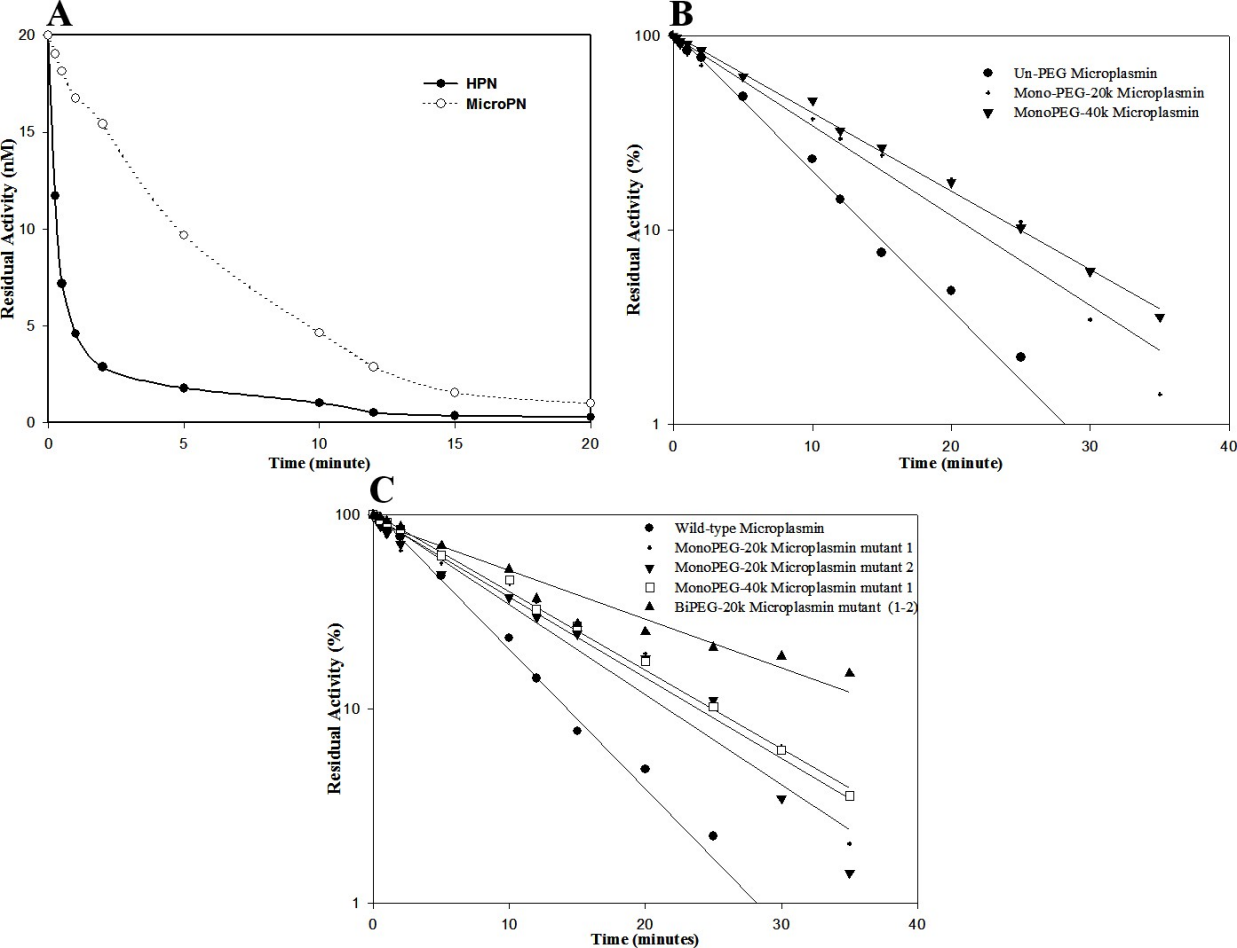
Activity inhibition analysis of wild-type and PEGylated microplasmin analogs. Inhibition kinetics were performed by adding microplasmin analogs (20nM) and antiplasmin (60nM, Calbiochem) to cuvettes containing 100mM sodium phosphate, pH 7.2 and incubating at 25°C for the time intervals ranging from 15sec-30 min as described under “Materials and methods”. Changes in absorbance were recorded at 405nm after the addition of 0.5 mM Chromozym PL. The residual enzyme activity was measured at different intervals from the slope of the curve and plotted as log % residual activity *versus* time. Panel (A) shows the time-dependent inhibition of wild-type microplasmin by α_2_-antiplasmin; In Panel (B) residual activity of mono-PEGylated mutants (different PEG sizes) has been shown; Panel (C) shows the comparative analysis of residual activities of mono-PEGylated and di-PEGylated mutants with different PEG sizes.

Time-dependence of wild-type microplasmin inhibition by α_2_-antiplasmin and its comparison with the PEG mutants was then studied. It was observed that PEGylated microplasmin analogs could retain their activity for significantly longer periods of time as compared to their unmodified counterparts (Table 5). The site-specifically PEGylated thiol mutants of microplasmin exhibit a significantly reduced inhibition rate relative to the wild type microplasmin (Fig 8C).

**Table 5:** *In vitro* half-life of inactivation of PEGylated Micro-plasmin and its un-PEGylated form by α_2_-antiplasmin.

Microplasmin variant	*In vitro* Half-life
Wild-type Microplasmin	5 ±1 min
mono-PEGylated microplasmin mutant (20kDa PEG)	6.5 ± 1.5 min
mono-PEGylated microplasmin mutant (40kDa PEG)	8 ±1.5 min
di-PEGylated microplasmin mutant (20kDa-20kDa PEG)	10 ± 1.5 min

In addition, the correlation of the number of conjugation sites, size of PEG group and their effect on α_2_-antiplasmin inhibition was also studied. The comparative *in vitro* half-lives of mono-PEGylated as well as di-PEGylated microplasmin mutants are presented in Table 5. It can be seen that di-PEGylation i.e. attachment of two simultaneous 20kDa-PEG groups at two different sites in microplasmin molecule contributed to its relatively longer activity compared to the mono-PEGylated ones.

## Discussion

The ability to modify protein structure away from the active site expands the realm of possibilities for preventing unwanted molecular interactions near the active site of an enzyme especially where relatively distant exosites are targeted. The rationale behind the present study was to investigate the effect of site selective PEGylation of microplasmin through a protein engineering approach.

The surface exposed loops among various serine proteases are considered to be important for their selective interactions with substrates and inhibitors [55, 64]. The docking models obtained from GRAMM-X Protein-Protein Docking Web Server v.1.2.0 [56] using available three dimensional structural information of murine antiplasmin as well as human plasminogen catalytic domain (PDB ID. 1DDJ) [55] were used to interpret interacting residues preferably lying on loop structures of micro-plasminogen. Residues were chosen by keeping it in mind that selected sites are distant from the catalytic site as well as the native cysteines of protein involved in disulfide linkage so that there is expected to be little interference with the fibrinolytic abilities. Absence of any free intrinsic cysteine in natively folded micro-plasminogen [68], offered a unique opportunity to strategically incorporate an unpaired cysteine into the micro-plasminogen for PEG-coupling. Therefore, microplasmin modification with a thiol-reactive PEG was done at specific sites in the latter, selected on the basis of structural features that may lend themselves to a steric inhibition by the PEG groups and their possible propensity to occupy solvent/intermolecular spaces during interactions between the protease and its inhibitor. The PEG mutants were generated by site-directed cysteine substitution mutagenesis, and selecting the mutants that preserve their biological activity. The characterization data of PEGylated microplasmin mutants suggests that any structural perturbation due to PEGylation is probably subtle. In contrast, the hydrodynamic radii of the PEGylated microplasmin are likely altered considerably according to the PEG chain length, as is well recognized in literature [69, 70]. Our results of DLS analysis of PEG-protein conjugates are quite concordant with the theoretical values of hydrodynamic radii of standard PEG groups as mentioned by Dong *et al*. [71]. However, a smaller hydrodynamic radius for di-PEGylated (20kDa at each site) conjugate as compared to the corresponding mono-PEGylated (20kDa) conjugate is indicative of a more compact structure, which could be due to more sterically restrained action/interaction with solvent owing to adjacent placement of two PEG chains in the loop.

Protein PEGylation may have important consequences on macromolecular recognition and the immunogenic response [72, 73]. A possible explanation of the resistant behavior of PEGylated microplamins may be the properties of steric hindrance resulting in a slower complexation between the complementing proteins. However, the interesting fact that this inhibition is not irreversible, but apparently only a kinetic one (since after the delay full native-like inhibition is seen) is possibly due to the sterical interference of PEG chains at critical protein-protein contacts [74]. In previous studies by Yang *et al*., in antibodies, physical hindrance by the bulky and mobile PEG moiety appeared to retard the association of PEGylated scFv with TNF-α. Based on plasmon-BIAcore analysis, where slower association rates with increasing mass of conjugated polymer were observed in the PEG conjugated scFv relative to the unmodified scFv [73]. Also, the available data of site-specific conjugation of 20kDa PEG to scFv demonstrated reduced apparent affinity of approximately 5-fold [75]. Further evidence provided by Kerwin *et al*., suggests that the decreased affinity of covalently bound PEG at N-terminus of sTNF-RI for free polymer could be ascribed to physical hindrance caused by bound PEG either shielding the binding site from the solvent or preventing/delaying a close approach of the polymer to the protein [72]. Furthermore, as observed in PEG-IFN, branched PEG conjugates displaying smaller size than the conjugates with linear PEGs of the equivalent molecular mass provided more pronounced shielding effect [76].

Thus, in the appropriate scenario, even the incorporation of relatively non-rigid PEG incorporation may not merely prolong renal/metabolic clearance rates, but at a molecular level, help to minimize/modulate protein-ligand intermolecular interactions, without removing them completely unlike more rigid groups. The results obtained in the present study indeed suggest that appropriately positioned flexible PEG moieties might be used to sterically interfere with the cognate recognition of microplasmin- α_2_-antiplasmin interacting sites, and consequently, slow the inhibitory reaction, unlike, say, a more rigid group/disruptive mutation which would tend to create a greater (and unwanted) structural alteration. It may be mentioned that PEGylation in general increases *in vivo* half-life of the altered molecule, a dramatically real-life useful instance being (among a large number of such examples) the creation of highly potent, long-acting GM-CSF analogs with up to 47-fold longer circulating half lives compared to wild type GM-CSF [77]. In contrast, in the present case, such an overtly increased *in vivo* half-life *per se* would have no beneficial outcome if it did not actually have an improved bio-activity survival as well. Thus, in the present work, PEGylation has successfully been utilized as a protein engineering tool to alter and improve the functioning of a therapeutic molecule, as opposed to mere survival time. Moreover, the present study suggests that the cumulative (additive) shielding effect of PEGs might be exploited further through a higher order of modification i.e. triple- or quadruple-site mutations that might not disturb the functioning of the molecule, but ones where a greater synergy affecting the interaction between α_2_ -antiplasmin and the modified plasmin derivatives, in a much more significant manner than observed with the single- and double-site mutants, is engendered.

Overall, the present study shows a potentially useful approach to obtain promising leads based on human Micro-plasmin that may be of clinical use in the near future, especially in thrombolytic therapies for ischemic stroke and also, possibly, intra-ocular applications [78].

## Conclusion

The present study illustrates the effect of targeted covalent grafting of PEG chains on human microplasminogen so as to slow the antiplasmin mediated inhibition of its activated form microplasmin. We have identified some functional hot spots in microplasminogen that allow effective attachment of PEG moieties to the surface of the microplasmin without dramatically affecting its intrinsic enzymatic activity. The experiments suggest that physical steric hindrance caused by the relatively mobile but appropriately placed PEG group affect the association of PEGylated microplasmin with α2 –antiplasmin when attached at these site/s, without any marked alteration of the former’s fibrinolytic potency. Overall, the outcome of the present investigation emphasizes that microplasmin interactions with antiplasmin can be inhibited even by the non-rigid PEG polymer through sterically effective positional placements in the former.

We suggest that the ability to modify sites away from the epitopes critical for bioactivity expands the realm of possibilities for preventing unwanted macromolecular interactions. However, further studies are likely to reveal a greater understanding of the substrate-inhibitor interaction mechanism of PEGylated proteins in order to enhance their biological performance and overall therapeutic outcomes by their use.
